# Peripheral Voltage-Gated Cation Channels in Neuropathic Pain and Their Potential as Therapeutic Targets

**DOI:** 10.3389/fpain.2021.750583

**Published:** 2021-12-13

**Authors:** Sascha R. A. Alles, Peter A. Smith

**Affiliations:** ^1^Department of Anesthesiology and Critical Care Medicine, University of New Mexico School of Medicine, Albuquerque, NM, United States; ^2^Department of Pharmacology, Neuroscience and Mental Health Institute, University of Alberta, Edmonton, AB, Canada

**Keywords:** Na_v_1.3, Na_v_1.7, Na_v_1.8, Ca_v_3.2, K_v_7.2/7.3, dorsal root ganglia (DRG), primary afferent, allodynia

## Abstract

The persistence of increased excitability and spontaneous activity in injured peripheral neurons is imperative for the development and persistence of many forms of neuropathic pain. This aberrant activity involves increased activity and/or expression of voltage-gated Na^+^ and Ca^2+^ channels and hyperpolarization activated cyclic nucleotide gated (HCN) channels as well as decreased function of K^+^ channels. Because they display limited central side effects, peripherally restricted Na^+^ and Ca^2+^ channel blockers and K^+^ channel activators offer potential therapeutic approaches to pain management. This review outlines the current status and future therapeutic promise of peripherally acting channel modulators. Selective blockers of Na_v_1.3, Na_v_1.7, Na_v_1.8, Ca_v_3.2, and HCN2 and activators of K_v_7.2 abrogate signs of neuropathic pain in animal models. Unfortunately, their performance in the clinic has been disappointing; some substances fail to meet therapeutic end points whereas others produce dose-limiting side effects. Despite this, peripheral voltage-gated cation channels retain their promise as therapeutic targets. The way forward may include (i) further structural refinement of K^+^ channel activators such as retigabine and ASP0819 to improve selectivity and limit toxicity; use or modification of Na^+^ channel blockers such as vixotrigine, PF-05089771, A803467, PF-01247324, VX-150 or arachnid toxins such as Tap1a; the use of Ca^2+^ channel blockers such as TTA-P2, TTA-A2, Z 944, ACT709478, and CNCB-2; (ii) improving methods for assessing “pain” as opposed to nociception in rodent models; (iii) recognizing sex differences in pain etiology; (iv) tailoring of therapeutic approaches to meet the symptoms and etiology of pain in individual patients *via* quantitative sensory testing and other personalized medicine approaches; (v) targeting genetic and biochemical mechanisms controlling channel expression using anti-NGF antibodies such as tanezumab or re-purposed drugs such as vorinostat, a histone methyltransferase inhibitor used in the management of T-cell lymphoma, or cercosporamide a MNK 1/2 inhibitor used in treatment of rheumatoid arthritis; (vi) combination therapy using drugs that are selective for different channel types or regulatory processes; (vii) directing preclinical validation work toward the use of human or human-derived tissue samples; and (viii) application of molecular biological approaches such as clustered regularly interspaced short palindromic repeats (CRISPR) technology.

## Introduction

Whilst opioids are extremely effective in managing deep and nociceptive pain, the drugs available for treatment of neuropathic pain display limited effectiveness (1, 2). Sites of action of anti-allodynic agents such gabapentinoids, tricyclic antidepressants, and noradrenaline-serotonin uptake inhibitors such as duloxetine or venlafaxine reside predominantly within the spinal cord and at other central loci (2–5). Because the persistence of aberrant and spontaneous activity in injured peripheral neurons is imperative for the development and persistence of many forms of neuropathic pain (2, 6–14), the peripheral nervous system offers a range of actual and potential drug targets. It has been argued that targeting the peripheral nervous system with substances that do not readily cross the blood-brain barrier, may circumvent the dose-limiting side effects seen with centrally acting agents (15). For example, adverse centrally-mediated effects of gabapentin include dizziness, somnolence, fatigue, ataxia, and nystagmus (16). This review thus outlines the current status and future promise of peripherally-acting agents; focusing on those that interact with cation channels in primary afferent neurons.

Peripheral nerve injury promotes Wallerian degeneration of severed axons, Schwann cell activation and the generation and release of chemokines, cytokines, and growth factors. These sensitize sensory nerve endings, attract macrophages and lymphocytes, alter gene expression, promote post-translational modification of proteins and alter ion channel function (17–23). The activity and/or expression of voltage-gated TTX-sensitive Na^+^ channels, voltage-gated Ca^2+^ channels, ASIC channels, TRP channels, and HCN channels is increased (24–27) whereas that of K^+^ channels is decreased (28). These peripheral ion channels thus present a viable target for therapeutic intervention (24, 28) as alterations in their activity underlies the increased excitability of primary afferents (11, 12, 29–35). In the interest of brevity, this review is confined to description of injury-induced changes in voltage-gated cation channels in primary afferent neurons and their potential as therapeutic targets. Information on ligand-gated channels which includes purinergic P2X3 channels, acid sensing ion channels (ASIC), and various types of TRP channel may be found in recent publications and reviews (3, 25, 36–40).

A summary of viable therapeutic approaches to the management of neuropathic pain by modulation of function or expression of voltage-gated cation channels is presented in Table 1.

**Table 1:** Potential and actual therapeutic candidates.

**Channel type**	**Drug**	**Mechanism of action**	**Status**
**Voltage-gated sodium channels**			
**Na**_**v**_ **1.3**	• miR-384-5p • miR-30b • miR-96	Negative regulation of the SCN3A gene for Na_v_1.3 (41–43).	Not yet tested in the clinic
	Diphenylmethyl amide adducts of an aryl sulphonamide series (44)	Channel block	Not yet tested in the clinic
**Na**_**v**_ **1.7**	Lacosemide	Inactivated state blocker	Safe and effective, in a randomized, placebo-controlled, double-blind, crossover-design study of Na_v_1.7 related small fiber neuropathy (45)
	PF-05089771	Inactivated state blocker	Failed to reach therapeutic end point in a diabetic neuropathy trial (46)
	CNV1014802 (vixotrigine or raxatrigine)	Inactivated state blocker	Trial ongoing for effectiveness in trigeminal neuralgia (47).
	Natural and chemically modified toxins such as JNJ63955918 • JzTx-V • PnTx1 • GpTx-1 • ProTx-11, μ-conotoxin KIIIA • μ-TRTX-Tp1a (Tp1a) • Tap1a • Tap1a-OPT1	Most of these toxins are gating modifiers	High affinity and selectivity of various toxins for Na_v_1.7 has been demonstrated (48). None as yet have entered clinical trials. Tap1a also blocks Cav3.2 channels (49). Structural modification of Tap1a may produce especially potent and effective agents (50)
	Low dose opioids in combination with Na_v_1.7 blockers	Augmentation of opioid contribution to effectiveness of Na_v_1.7 blockers (51–53)	No clinical information presently available
	“LATER” (long-lasting analgesia *via* targeted *in vivo* epigenetic repression) technology	CRISPR epigenetic technology to suppress Na_v_1.7 expression	Encouraging results found in hiPSC (54, 55).
	Carbamazepine	Channel block	Use primarily restricted to trigeminal neuralgia (56)
**Na**_**v**_ **1.8**	A803467 PF-01247324	Small molecule pore blockers	Not yet tested in clinic
	VX-150	Prodrug metabolized to small molecule pore blocker	Clinical trial ongoing (57)
	Tanezemab	Monoclonal antibody directed at nerve growth factor	Trials in several pain states have brought forth encouraging results (58)
Multiple actions on Na^+^ channels	Cyclic peptides derived from the structures of natural product channel blockers μ-conotoxin KIIIA and (PnTx1) *Phoneutria nigriventer* toxin 1 (59)	Channel block	Ongoing studies seek to improve toxin selectivity
	Lidocaine patch	Inactivated state blocker	In clinical use (1)
	Cationic local anesthetics combined with TRPV1 activators (60–62)	Local anesthetic effect achieved selectively in TRPV1 expressing neurons by anesthetic permeation of TRPV1 channels	Preclinical research is ongoing, but no reports of clinical investigations
**Voltage-gated potassium channels**			
**K**_**v**_**1.1**. *Delayed Rectifier K^+^ channels*	2-fluorophenyl glycine	Direct channel activator (63)	Under consideration for use in episodic ataxia type 1, as yet untested in pain models
**K**_**v**_**1.2** *Delayed Rectifier K^+^ channels*	Suberoylanilide hydroxamic acid (Vorinostat)	HDAC2 inhibitor may attenuate pain by increased expression of K_v_1.2 and by other mechanisms (64–66)	Clinically approved anti neoplastic agent not yet evaluated in cancer or neuropathic pain
**K**_**v**_**2.1, K**_**v**_**2.2** *Delayed Rectifier K^+^ channels*	Activators of associated Kv1.9 silent subunits	Formation of hetero—tetramers (Kv1.9–2.1–2.2) may increase overall channel conductance (67)	Suitable compounds or methodology not yet developed
**K**_**v**_**7.2** *KCNQ2 or M-channels*	Retigabine	M-channel opener	Failed to meet its efficacy endpoint in post herpetic neuralgia (68)
	Flupirtine	M-channel opener	Withdrawn because of toxicity issues
	SCR 2682	K_v_7.2 opener which also increases KCNQ2 mRNA and K_v_7.2 protein expression (69, 70)	Mechanism of action yet to be determined, not yet ready for clinical trials
	Mallotoxin Isovaleric acid (E)-2-dodecenal	Natural products that act as K_v_7.2/7.3 activators (71, 72)	Effective in animal models of epilepsy, efficacy in pain models not yet examined
**K**_**v**_**4** *A-channel*	NS5806	Modulation of K_v_ channel activity by interactions with KChips (73, 74)	Attenuates cold allodynia in a model of trigeminal neuralgia (75)
**K**_**ir**_**6.2** *K_*ATP*_ channels*	Diazoxide Minoxidil	K_ATP_ channel openers (76–79)	Despite efficacy in neuropathic pain models their use in the clinic has not been advocated.
Multiple actions on K^+^ channels **K**_**v**_**7.2** *KCNQ2 or M-channels* **K**_**v**_**1.4** *A-channel* **K**_**Ca**_**1.1** *BK Ca^2+^ sensitive K^+^ channel*	BIX01294 UNC0638	Inhibition of histone methyltransferase G9a (80, 81)	Histone methyltransferase inhibitors are being developed as antineoplastic agents, use in clinical pain yet to be established.
**Voltage-gated calcium channels**			
N-type voltage-gated Ca^2+^ channels (**Ca**_**v**_**2.2**)	Ziconotide (Synthetic ω-conotoxin MVIIA)	Channel block	Administered intrathecally when other treatments fail (82)
	• Small molecule blockers • ZC88 • A1264087 • TROX-1 • (83–87)	Channel block	No clinical data yet available
	Clonidine	Channel block *via* α2 adrenoceptor and Gi/o interaction	Only effective in small subgroups of patients (88–92).
	Gabapentinoids	Affect Ca_v_2.2 channel trafficking and association with release machinery both peripherally and centrally (93–95)	Classical anti allodynic agent (1), but only effective in 31% of patients (96)
	CNCB-2	Bifunctional, permanently charged molecule blocks Ca_v_2.2 and Na_v_1.7. (97)	Yet to be examined in animal models of neuropathic pain
T-type voltage-gated Ca^2+^ channels (**Ca**_**v**_**3.2**)	Ethosuximide	Classical T-current blocker and anticonvulsant	Clinical results in pain are disappointing (98)
	Suramin	Shows analgesic activity in neuropathic and inflammatory pain models by prevention of action of deubiquitinase, USP5(99, 100)	No clinical data
	• TTA-P2 • TTA-A2	Small molecule blockers effective in animal models	No clinical data
	• Z 944 • ACT709478	Small molecule blockers	Promising preliminary data from clinical trials (101)
	Tap1a	Toxin derived from tarantula venom	Also blocks Nav1.7 and shown to be effective in murine model of irritable bowel syndrome (49)
BK current, T current, Ca_v_2.2, Na_v_1.8	Cannabinoids	(102–105)	Considerable discussion in the literature relates to the efficacy of cannabinoids in neuropathic pain (102, 106–109)
**Interactions with transduction mechanisms that control nociceptor excitability**			
	Cercosporamide	MNK 1/2 Inbitor	Suppresses pain in murine models (110) and is approved for management of pain in rheumatoid arthritis
	Vorinostat	Histone methyltransferase inhibitor	Alleviates pain in a bone cancer model (66)

## Voltage-Gated Na^+^ Channels

Injury-induced increases in Na^+^ channel function were first described over 20 years ago (111–113). They reflect altered expression of channel protein and/or its accessory subunits, altered trafficking or post-translational modification and/or modulation (114, 115).

The genetic and structural definitions of Na_v_1.1–Na_v_1.9 channel subtypes was also established many years ago (116–118) and this has led to a mechanistic and molecular understanding of injury-induced changes (8, 114). This has paved the way for selective targeting of TTX-sensitive Na_v_1.3, 1.6, and 1.7 channels and TTX-resistant Na_v_1.8 channels as these are particularly important in the generation and maintenance of neuropathic pain (114, 119–122).

As described below, different Na_v_ channel subtypes in different neuronal populations are involved in different types of neuropathic and nociceptive pain (114, 123–125).

### Expression and Therapeutic Modulation of TTX-Sensitive Na^+^ Channels

#### Role of Na_v_1.3 in Neuropathic Pain

Na_v_1.3 channels were previously known as type III Na^+^ channels. They are TTX-sensitive products of the *SCN3A* gene and are found in neurons and cardiac myocytes with the highest level in embryonic and early postnatal animals (117, 126, 127). In DRG neurons, they exhibit rapid recovery from inactivation or “repriming,” thereby enhancing repetitive discharge (128). Their involvement in neuropathic pain is supported by the attenuation of allodynia seen with intra-ganglionic injection of adeno-associated virus expressing small hairpin RNA targeting Na_v_1.3 (129). Nerve injury upregulates and promotes re-expression of Na_v_1.3 in adult DRG neurons (127, 130, 131) as well as in spinal dorsal horn and thalamus (132, 133). This may reflect removal of suppression of the *SCN3A* gene by microRNAs such as miR-384-5p, mir-96 and/or miR-30b suggesting that their targeted delivery may be of use in pain management (41–43).

#### Pharmacological Manipulation of Na_v_1.3

Because Na_v_1.3 is mainly present in embryonic and early neonatal animals and because nerve injury promotes selective upregulation of Na_v_1.3 in nociceptive pathways of adults, there is considerable interest in developing Na_v_1.3 blockers. Structure activity studies starting with a diphenylmethyl amide adduct of an aryl sulphonamide has led to the development of compounds with good selectivity for Na_v_1.3 as well as favorable pharmacokinetics (44).

#### Role of Na_v_1.6 in Neuropathic Pain

Na_v_1.6 is another TTX-sensitive Na^+^ channel. It is the product of the *SCN8A* gene (117) and was previously known as PN4. Na_v_1.6 channels are expressed along the whole length of sensory unmyelinated axons (134) and are clustered at nodes of Ranvier in myelinated fibers where they participate in “saltatory” conduction (135).

The observation that knockout of Na_v_1.6 reduces injury-induced pain behaviors and sensory neuron excitability (136–138) implicates it in the etiology of neuropathic pain. It has recently been implicated in a model of vincristine-induced chemotherapy induced peripheral neuropathy (CIPN) and allodynia (139) and is upregulated in the DRG in a model of diabetic neuropathy (140). These findings are corroborated by the description of a gain-of-function mutation in Na_v_1.6 in a case of trigeminal neuralgia (141). Since its role in in pain etiology was established relatively recently (114, 142), there have been as yet no attempts to modulate Na_v_1.6 channel activity either in animal models or in the clinic.

#### Role of Na_v_1.7 in Neuropathic Pain

The TTX-sensitive Na_v_1.7 channel is involved in a multiplicity of neuropathic and nociceptive pain states (8, 48, 54, 114, 123, 143–146). It is the product of the *SCN9A* gene and was previously known as PN1. Na_v_1.7 is the dominant voltage-gated Na^+^ channel in peripheral sympathetic neurons and in all types of DRG neuron (117, 147). Its expression extends from peripheral nerve endings in the skin and viscera to primary afferent terminals in the dorsal horn (148) where it is especially concentrated (147). Na_v_1.7 is preferentially expressed in small diameter nociceptors including both the CGRP-positive subcategory and the non-peptidergic subcategory that bind the plant lectin IB4 from *Griffonia simplicifolia* (114). It is also found in olfactory sensory neurons, magnocellular neurosecretory cells of the hypothalamic supraoptic nucleus and in vagal afferents (51, 149–151). Because it is not found to any great extent in vital non-neuronal tissue such as heart or skeletal muscle (114, 147), Na_v_1.7 represents a specially attractive target for therapeutic manipulation. Although it is found in pancreatic alpha and beta cells it may be inactivated at their normal resting potential (152).

Immunohistochemical studies first demonstrated Na_v_1.7 upregulation in severed axons within human painful neuromas (122, 153) and Na_v_1.7 has been shown to be necessary for the release of the pain modulator substance P from primary afferent terminals (124).

Despite this, Na_v_1.7 does not appear to be involved in all manifestations of neuropathic pain. For example oxaliplatin-induced pain and cancer-induced bone pain do not require the presence of Na_v_1.7 or the Na_v_1.8-positive nociceptors in which Na_v_1.7 is enriched (123). By contrast, paclitaxel-induced CIPN involves the direction of Na_v_1.7 to cell membranes and axons of primary afferent fibers (154). Also, neuropathic pain produced by constriction injury (CCI) is abolished when Na_v_1.7 is *selectively* deleted in murine sensory neurons and although spinal nerve transection or tight ligation (SNL) also produces cold and mechanical allodynia this is not affected by *selective* knockout of Na_v_1.7 in DRG neurons. By contrast, knockout of Na_v_1.7 in both sympathetic and sensory fibers attenuates both forms of allodynia (123). This is because SNL involves sprouting of Na_v_1.7 expressing perivascular sympathetic fibers (155, 156) and their ectopic interaction with DRG neurons (157–159).

Patients with a rare, chronic pain conditions such as primary erythromelalgia or paroxysmal extreme pain disorder exhibit gain of function mutations in *SCN9A* (8, 146, 160–163). As of 2019, 30 mutations in *SCN9A* genes had been described in inherited erythromelalgia and 13 in paroxysmal extreme pain disorder (114). In the case of inherited erythromelalgia, isoleucine 848 is replaced by threonine. This I848T mutation increases the amplitude of current produced by Na_v_1.7 in response to slow, small depolarizations as a result of a hyperpolarizing shift in activation and slowed deactivation (161). Recently, protein kinase C has been found to be responsible for the phosphorylation of T848 found in mutant channels and this accounts for the shift in activation (164). Meents et al. (165) have differentiated human induced pluripotent stem cells (hiPSC) from erythromelalgia patients into sensory nociceptors. This will provide an extensive supply of human nociceptors for further study of erythromelalgia. Mutations seen in Na_v_1.7 channels of erythromelagia patients also occur in those with paroxysmal extreme pain disorder with an additional suppression of fast inactivation (163). Gain of function mutations of *SCN9A* also worsen neuropathic pain in a small cohort of patients with painful diabetic neuropathy (166).

Although some patients with small fiber neuropathy display the I228M gain-of-function mutation in Na_v_1.7, a pain phenotype does not appear until they reach adulthood (167). Expression of this same mutation in mice promotes increased DRG excitability without the appearance of a measurable pain phenotype. It is suggested that some compensatory mechanism may restrain the development of pain in the mouse model and the possible existence of a similar process in humans may delay the development of a pain phenotype until adulthood (168).

Patients with a rare congenital insensitivity to pain (CIP) express a loss of function mutation in Na_v_1.7 (169) and global knockout of Na_v_1.7 in mice recapitulates this human phenotype (170). Differentiation of hiPSC's from CIP patients into sensory nociceptors, produced cells where Na_v_1.7 was appropriately expressed and trafficked to the cell membrane. Since these cells failed to respond to depolarizing stimuli, CIP can be attributed to changes in the function of the channels *per se* rather than defects in their expression or trafficking (54). These results also provide new evidence for a role of Na_v_1.7 in human nociception. As of 2019, 26 mutations in *SCN9A* have been reported to contribute to CIP.

In addition to its role in controlling neuronal excitability and neurotransmitter release, Na_v_1.7 directly or indirectly affects gene expression (51, 52, 171). Na_v_1.7 deletion, leads to upregulation of *Penk* mRNA for the enkephalin precursor proenkephalin in DRG as well as met-enkephalin protein. Since a similar effect is seen with TTX, the upregulation of endogenous opioid function may be contingent on decreased levels of intracellular Na^+^ (52). These authors also showed that blockade of opioid receptors with naloxone reduces the analgesia seen in both male and female Na_v_1.7-null mutant mice and in a human patient with Na_v_1.7 dependent congenital insensitivity to pain [see also (51)]. The relationship between increased opioid function and decreased Na_v_1.7 function is supported by the observation that the analgesic effect of a selective Na_v_1.7 blocker, μ-theraphotoxin-Pn3a (from the tarantula *Pamphobeteus nigricolor*), is augmented by administration with sub-effective doses of opioids or with an enkephalinase inhibitor (172). Further analysis of this effect showed that Na_v_1.7 knockout mice have normal peripheral nociceptor activity but synaptic transmission from nociceptor central terminals is greatly reduced in an opioid-dependent fashion. Analgesia was reversed substantially by central but not peripheral application of opioid antagonists (51). These authors thus concluded inhibition of neurotransmitter release is the principal mechanism of analgesia in mouse and human Na_v_1.7-null mutants.

Second order sensory neurons in the spinal dorsal horn express few transcripts of Na_v_1.7 mRNA. Despite this, immunoreactivity for channel protein is abundant yet is reduced following rhizotomy (173). This suggests that sensory neurons are the source of Na_v_1.7 in spinal dorsal horn neurons and that intercellular transport of the protein occurs between these two neuronal populations. This conclusion was supported by the observation that selective deletion of Na_v_1.7 in peripheral neurons reduced the intrinsic excitability of dorsal horn neurons.

#### Pharmacological Manipulation of Na_v_1.7

Although it may not be involved in all types of neuropathic pain (123) it is absence from non-neuronal tissue such as heart or skeletal muscle (114, 147). Na_v_1.7 is therefore clearly an attractive target for therapeutic intervention (48, 114, 145, 154, 174). Moreover, the anticonvulsant lacosamide, which is an inactivated state blocker of Na^+^ channels (175, 176) has been found to be safe and effective, in a randomized, placebo-controlled, double-blind, crossover-design study of Na_v_1.7 related small fiber neuropathy [(45), Table 1]. Also, the effectiveness of carbamazepine which is used to treat trigeminal neuralgia (56) may in part reflect its affinity for Na_v_1.7 (177).

There is also considerable interest in various sulfonamide analogs which display selectivity toward Na_v_1.7 and are effective in pain mitigation in animal models [(178–183); see Table 1]. Therapeutic concentrations of the inactivated state blocker PF-05089771 increase the rheobase of control neurons, but not that of Na_v_1.7 knock-out neurons. Despite this selectivity for Na_v_1.7 and its effectiveness in animal models *in vivo* (54), a clinical study of PF-05089771 in subjects with painful diabetic peripheral neuropathy failed to meet defined efficacy criteria (46).

Another broad spectrum non-sulfonamide Na_v_ blocker, vixotrigine, which was previously known as raxatrigine, or CNV1014802, BIIB074, or GSK-1014802 (184), has shown effectiveness in animal models of Na_v_1.7-dependent pain. Its safety in human patients has been established (185). A phase III clinical trial for effectiveness in trigeminal neuralgia and phase II trial for small fiber neuropathy are presently ongoing (47).

#### Na_v_1.7 and Natural Toxins

Another approach to therapeutic modulation of Na_v_1.7 activity involves potential use and/or structural modification of natural toxins (48, 186–190). These are typically gating modifiers as opposed to simple pore blockers so some natural toxins increase channel function whereas others attenuate it [(48); Table 2]. Starting points include the cone snail toxin, μ-conotoxin KIIIA, and PnTx1 (*Phoneutria nigriventer* toxin 1) from a Brazilian spider. Although structure activity studies of small cyclic peptides derived from the structure of these toxins has not as yet revealed Na^+^ channels subtype ligands, the analgesic effect of many of the ligands involves modulation Na_v_1.7 channel function. This result was achieved by observing attenuation of pain produced by the Na_v_1.7 selective activator α-scorpion toxin OD1 [(191); Table 2]. Further modifications of small cyclic peptides may reveal more subtype selective ligands with appropriate pharmacokinetics *in vivo* and improved bioavailability (59).

**Table 2:** List of toxins that modulate Na_v_1.7 channel activity.

**Toxin name**	**Abbreviation(s)**	**Biological source**
**Inhibitors of channel function**		
μ-theraphotoxin-Pn3a	Pn3a	Tarantula *Pamphobeteus nigricolor*
*Phoneutria nigriventer* toxin 1	PnTx1	Brazilian spider *Phoneutria nigriventer*
*Grammostola porter* Toxin 1	GpTx-1	Rose hair or Chilean tarantula *Grammostola porteri*
Jingzhaotoxin-V	JzTx-V	Chinese tarantula *Chilobrachys jingzhao*
μ-theraphotoxin-Tp1a	μ -TRTX-Tp1a (also known as Tp1a or ProTX-III)	Peruvian green velvet *Thrixopelma pruriens*
*Theraphosa apophysis* Toxin 1a	Tap1a	Venezuelan tarantula *Theraphosa apophysis*
*Davus fasciatus* Toxin 1a	μ-TRTX-Df1a (also known as Df1a)	Costa Rican tiger rump tarantula *Davus fasciatus*
Huwentoxin-IV	HWTX-**IV**	Chinese bird spider *Haplopelma schmidti*
Hainantoxins	HNTX I and III	Chinese bird spider *Ornithoctonus hainana*
**Activator of channel function**		
α-scorpion toxin OD1	OD1	Scorpion *Odonthobuthus doriae*

Studies and modification of arachnoid toxins which display natural selectivity toward Na_v_1.7 may also lead to development of effective agents (190). As listed in Table 2, there are several examples.

Venom from the tarantula *Grammostola porteri* contains the 34-residue peptide, GpTx-1, with high and selective affinity for Na_v_1.7 (IC_50_ = 10 nM). Structural modifications of this peptide led to the identification of [Ala5, Phe6, Leu26, Arg28] GpTx-1 (also known as GpTx-1-71) IC_50_ = 1.6 nM (192). Both peptides exert powerful antinociception in mouse models of acute, visceral, inflammatory and neuropathic pain without impairment of motor co-ordination or development of tolerance (144). Another modified toxin derived from JzTx-V (from venom of the Chinese tarantula *Chilobrachys jingzhao*) has a 100-fold improved efficacy compared to GP-Tx-1-71 (193).

Studies of the venom from the Peruvian green-velvet tarantula *Thrixopelma pruriens* revealed a 33 residue peptide termed μ-TRTX-Tp1a (Tp1a or ProTx-III) with high selectivity and affinity for Na_v_1.7 (194). Unlike other spider toxins that inhibit the function of Na_v_ channels, Tp1a inhibited hNaV1.7 without significantly altering the voltage-dependence of activation or inactivation. Like PnTx1, the analgesic effect of Tp1a was demonstrated by its ability to reverse spontaneous pain induced in mice by intraplantar injection of the Na_v_1.7 activator OD1 (194).

Recently another peptide toxin named Tap1a from the Venezuelan tarantula *Theraphosa apophysis* was shown to reverse colonic mechanical hypersensitivity in a mouse model of irritable bowel syndrome. The toxin's efficacy was shown to reflect selective targeting of Na_v_1.7 as well as the T-type Ca^2+^ channel Ca_v_3.2 (49).

High-throughput screening has also identified μ-TRTX-Df1a (Df1a) from the venom of the spider *Davus fasciatus* as an Na_v_ modulator. This 34-residue peptide inhibits responses mediated by Na_v_1.7 that is endogenously expressed in the human neuroblastoma cell line SH-SY5Y. It also inhibits T-type calcium (Ca_v_3.1 and Ca_v_3.3) currents and other Na_v_ currents expressed in HEK 293 cells but has no effect on the voltage-gated potassium channel [K_v_2.1; (195)]. Df1a is active *in vivo* and reverses the spontaneous pain behaviors induced by the scorpion venom Na_v_ activator OD1.

Other investigations have used the venom-peptide ProTX-II (Protoxin II) from the Peruvian green velvet tarantula (*Thrixopelma pruriens*) as a scaffold, to engineer a library of over 1,500 peptides. This identified JNJ63955918 as a potent, highly selective, closed-state Na_v_1.7 blocking peptide which induces insensitivity to pain that closely recapitulates key features of the Na_v_1.7-null phenotype seen in mice and humans (196).

More recently attention has been drawn to huwentoxin-IV, from the Chinese bird spider *Haplopelma schmidti*. Because it has high affinity for sodium channels it is an attractive scaffold for engineering Na_v_1.7-selective molecules and several new ligands with high affinity and selectivity have been identified (197).

Other natural products which block Na_v_1.7 channels include HNTX I and III from the spider *Ornithoctonus hainana* (198, 199), bulleyaconitine from *aconitum bulleyanum* plants (200) and the Japanese traditional medicine goshajinkigan (2, 201).

#### Clinical Status of Na_v_1.7 Blockers

In general, despite intensive pre-clinical studies with Na_v_1.7 blockers, tests of their efficacy in the clinic has yielded rather disappointing results [(48), Table 1] and to the best of our knowledge no studies of tarantula and other toxins in the clinic have appeared. Nevertheless, the continued study of toxins, small molecule blockers and monoclonal antibodies (202) should and will continue (2, 8). In particular, further structural modification of small molecule blockers such as CNV1014802 (vixotrigine) and PF-05089771 as well as chemical modification of natural toxins (48, 50) may provide a route to the development of more efficacious therapeutic entities. The tarantula toxin Tap1a shows particular promise as it appears to selectively target both Na_v_1.7 and Ca_v_3.2 (49).

Since the consequences of Na_v_1.7 blockade are mediated at least in part by endogenous opioids (51, 52), benefit may be obtained by combining small molecule blockers or toxins with low doses of opioids (48, 53).

The development of monoclonal antibodies and the delivery of the inhibitory micoRNA miR-182 (203) or modifiers of Na^+^ channel β subunits (204) may reveal additional therapeutic approaches. This approach may be especially attractive as three different types of β subunits are differentially and selectively expressed in small, medium, and large diameter DRG neurons (205, 206).

An approach that has proved particularly effective for targeting Na_v_1.7 uses CRISPR-dCas9 technology (clustered regularly interspaced short palindromic repeats) (55). Epigenome engineering platforms were introduced intrathecally in mice *via* adeno-associated viruses. A novel approach that prevented expression of Na_v_1.7 by editing a regulatory sequence successfully repressed Na_v_1.7 expression in lumbar DRG, reduced thermal hyperalgesia in inflammatory pain models and decreased tactile allodynia in the neuropathic pain models without affecting normal motor function. It is anticipated that this “LATER” (long-lasting analgesia *via* targeted *in vivo* epigenetic repression technology) might have therapeutic potential in management of persistent pain states. This is important in practical terms as chronic pain patents usually present in the clinic when they have suffered for many months. The technology can of course be easily modified to control expression of any potential or central drug target.

### Expression and Therapeutic Modulation of TTX-Resistant Na^+^ Channels

#### Role of Na_v_1.8 in Neuropathic Pain

The TTX-resistant Na_v_1.8 channel is predominant in small DRG neurons (124, 207–210) but its selective association with nociceptors has been questioned (211). It was originally known as SNS or PN3 and is encoded by the *SCN10A* gene (117). It is characterized by its high threshold for activation and its slow rate of inactivation at depolarized potentials (210). These properties enable it to generate a slow persistent inward current (212).

Although peripheral nerve injury attenuates Na_v_1.8 function in injured DRG neurons (213–215) it is thought to accumulate in uninjured neurons (216) and in neuromas that develop at sites of nerve injury (217). Selective blockade of Na_v_1.8 function promotes hypoalgesia (213), gain of function mutations of *SCN10A* in humans can promote painful neuropathy (218) and its optogenetic silencing in DRG attenuates neuropathic pain (219).

#### Pharmacological Manipulation of Na_V_1.8

The selective Na_v_1.8 blockers A803467 and PF-01247324 are being developed as potential antidysrhythmic agents (220). Although both are reported to attenuate allodynia in a rodent model (221, 222), they have yet to be used in clinical studies (223). Encouraging results have been seen with the pro-drug VX-150 which exhibits analgesic activity in healthy volunteers (57), but preclinical literature in support of these studies are not available online. The μO-conotoxins, MrVIA, MrVIB, and MfVIA block Na_v_1.8 and ongoing analysis seeks to increase their affinity by structural modifications (224).

Unlike the situation with Na_v_1.7, analgesia produced with blockade of Na_v_1.8 is not opioid-dependent (52) and may be attributable to decreased excitability of peripheral afferents and their central terminals (225).

Although the efficacy of the non-psychoactive cannabinoid, cannabidiol in management of neuropathic pain remains to be established (106), it was recently reported to decrease the excitability of DRG neurons by binding to the slow inactivated state of Na_v_1.8 channels (102).

Expression of Na_v_1.8 in peptidergic DRG neurons is controlled by nerve growth factor (NGF) (215) whereas its expression in non-peptidergic neurons is controlled by glial colony derived neurotrophic factor (GDNF) (226). This may account in part for the effectiveness of the NGF antagonist tanezumab in various pain states (58). In fact, its safety and efficacy in humans identifies tanezumab as one of more the promising new drug candidates for chronic and neuropathic pain (see Clinical Trials Government Identifiers: NCT02528188 and NCT02528188).

#### Role of Na_v_1.9 in Inflammatory Pain but Not in Neuropathic Pain

Na_v_1.9 is also TTX-resistant (227) and is encoded by the *SCN11A* gene. It was previously known as NaN. Unlike genes encoding other voltage-gated Na^+^ channels, murine *SCN11A* is only 75% identical to the human gene (114). Na_v_1.9 was previously known as NaN or SNS-2 (117) and because it inactivates extremely slowly, it is capable of producing a persistent inward current (228). This means that gain of function mutation of Na_v_1.9 causes *decreased* excitability because other voltage-gated Na^+^ channels are inactivated by persistent Na_v_1.9 mediated depolarization (8). In the peripheral nervous system, NaN/Na_v_1.9 was first detected in small DRG neurons of SNS/Na_v_1.8—null mice (228) where it is preferentially expressed in non-peptidergic neurons which bind the plant lectin IB4 (229). Channels are found in free nerve endings, along axons, in DRG cell bodies and in primary afferent terminals in spinal lamina II (*substantia gelatinosa*) (230). Unlike Na_v_1.7, sciatic injury reduces expression of mRNA and channel protein for Na_v_1.9 (231) and this may be attributable to loss of trophic support by GDNF (226). Since Na_v_1.9 knockout mice continue to display allodynia following nerve injury (232), this channel is unlikely to play a role in injury-induced neuropathic pain. This contrasts with the situation for inflammatory pain where a role for Na_v_1.9 is well-established (114, 232).

### Selective Modulation of Na^+^ Channels in TRPV1 Nociceptors

The local anesthetic, lidocaine acts in its cationic form to block all types of Na^+^ channels from the cytoplasmic side of the membrane. Although the topical application of lidocaine by means of a transdermal patch continues to be used in clinical pain management (1), disturbance of other aspects of sensory transmission by local anesthetics necessitates the development of more refined approaches. An ingenious approach has been used to selectively target lidocaine to TRPV1 expressing nociceptors (60, 233). The quaternary analog of lidocaine, QX314 is unable to permeate the cell membrane. It is therefore ineffective when applied extracellularly but is an effective local anesthetic when applied to the cytoplasmic side of the cell membrane. The pore of open TRPV1 channels is large enough to admit QX314, so their activation on nociceptors by capsaicin allows entry of QX314 and an anesthetic effect which is selective for this neuronal population. Although these findings have been repeated by others (234, 235) and the effectiveness of a more potent cationic anesthetic BW-031 described (61), this approach is yet to be exploited in a clinical situation.

## Voltage-Gated K^+^ Channels

It is well-established that decreased function of voltage-gated K^+^ channels contributes to injury-induced increases in peripheral nerve excitability and activity (28, 236–245). As with Na^+^ channels, K^+^ channel function can be modified by altered expression of channel protein and/or its accessory subunits, altered trafficking or post-translational modification or modulation. Also, the establishment of genetic and structural definitions of a broad variety of K^+^ channel types (246–249) has led to improved mechanistic understanding of injury induced changes. Although the selective targeting of K^+^ channels has so far been less rewarding than targeting of voltage-gated Na^+^ channels, potential targets include K_v_7.2 and the histone methyltransferase G9a which controls expression of several voltage-gated K^+^ channels, namely K_v_7.2, K_v_1.4 K_Ca_1.1 [(250), Table 1].

### Decreased Expression and Therapeutic Modulation of Delayed Rectifier K^+^ Channels

Sciatic nerve transection decreases functional expression of delayed rectifier K^+^ currents in DRG neurons (236–238, 251). Injury-induced changes may in part reflect post-translational processes such as phosphorylation, endocytosis and/or trafficking (245, 252, 253) that may be independent of any change in expression of K^+^ channel genes and their products as will be described in detail below. This possibility is underlined by the observation that delayed rectifier currents are substantially reduced in a rodent model of painful diabetic neuropathy but the mRNA levels for K_v_1.1, K_v_1.2, K_v_2.1, and K_v_2.2 are unchanged (254).

There are many types of delayed rectifier K^+^ channels in DRG neurons that assemble as hetero-tetramers or homo-tetramers of various K_v_1, K_v_2, and K_v_3 subtypes (28). Although most types of K_v_1 and K_v_2 channels are affected by peripheral nerve injury, their ubiquitous distribution in both excitable and non-excitable tissues restricts the therapeutic potential of substances that augment the activity of delayed rectifier K^+^ channels.

#### Role of K_v_1.1 in Neuropathic Pain

Protein and mRNA for K_v_1.1 is reduced in DRG following sciatic nerve injury (238, 245, 255) and this is associated with redistribution of channels away from nodal regions of A-δ fiber axons (245). Although expression of a dominant negative phenotype of K_v_1.1 causes allodynia in mice (256), certain glycine derivatives act as Kv1.1 channel openers (63), and substances have been identified that attenuate the time dependent inactivation of K_v_1.1 (257), its ubiquitous distribution in brain, heart, retina, skeletal muscle and pancreatic islets (247) may preclude the use of K_v_1.1 activators in pain management.

#### Role of K_v_1.2 in Neuropathic Pain

Knockdown of K_v_1.2 by siRNA induces mechanical and thermal hypersensitivity in naive rats (258). mRNA for K_v_1.2 is also downregulated in several neuropathic pain models (28, 238, 255, 259, 260), and overexpression of Kv1.2 impairs neuropathic pain but does not attenuate acute pain in rats (261). These findings correlate with injury-induced reduction of whole-cell K_v_1.2 current (260) and reduced channel protein expression as demonstrated by immunohistochemistry (261, 262) and/or immunoblot (245, 263).

Six different mechanisms have been hitherto suggested to underlie decreased K_v_1.2 expression in DRG after peripheral nerve injury.

- Altered expression of histone deacetylase2 (HDAC2) (263) by NF-κB p65-dependent transcriptional regulation (264).
- Increased expression of the canonical maintenance methyltransferase DNMT1 *via* a CREB (cAMP response element binding protein)—dependent process. Blockade of DNMT1 upregulation attenuates hyperexcitability in the injured DRG neurons and alleviated nerve injury-induced pain hypersensitivity (260, 265).
- A pathway involving the methyl-CpG-binding domain protein 1 (MBD1), which binds to methylated sequences of DNA and attracts the DNA methylation protein DNMT3a. Overexpression of MBD1 leads to spontaneous pain and evoked pain hypersensitivities in wild type mice (266, 267).
- Decreased expression of ten-eleven translocation methylcytosine dioxygenase 1 (TET1). This promotes DNA demethylation and its overexpression in the DRG of nerve injured animals alleviates pain hypersensitivities without altering acute pain (268).
- K_v_1.2 function may be controlled by the non-coding miniature RNA miR-137. Because it impairs K_v_1.2 function, experimental impairment of miR-137 function, rescues channel expression and function and attenuates allodynia in rats subject to CCI (258).
- A long non-coding RNA (Kcna2 antisense RNA) contributes to neuropathic pain by silencing the *KCNA2* gene and thereby reducing expression of K_v_1.2 in primary afferents (259).

#### Limited Feasibility of Pharmacological Manipulation of K_v_1.2

No small molecule activators of K_v_1.2 have been identified (118) and given their documented presence throughout the brain, in spinal cord, mechanoreceptors and proprioceptors, Schwann cells, the heart, vascular smooth muscle and retina (247), direct pharmacological manipulation of these channels is not a viable means of treatment of neuropathic pain. There are some reports of alleviation of pain in animal models by attenuation of HDAC2 action (64, 65) but these may reflect modulation of its actions in the spinal cord as well as upregulation of K_v_1.2 in the periphery. The HDAC inhibitor and antineoplastic agent, suberoylanilide hydroxamic acid (vorinostat) has been shown to alleviate pain in a bone cancer model (66) but to the best of our knowledge no trails of its efficacy in any form of neuropathic pain have as yet appeared.

#### Minimal Role of K_v_1.3, 1.5, and 1.6 in Injury- Induced Pain

These channels which also exhibit delayed rectification are expressed at relatively low levels compared to K_v_1.1 and 1.2 in naïve DRG (238, 245). mRNA for K_v_1.3 is decreased but that for K_v_1.5 and 1.6 is little affected by nerve injury (238, 255). In view of the relatively limited expression of these channels in DRG, augmentation of their function would not seem to be a desirable therapeutic strategy for pain mitigation.

#### A Role for K_v_2.1, 2.2, and K_v_9.1 in Injury-Induced Pain

Channel protein and mRNA are reduced by nerve injury as is K_v_2 whole-cell current comprising K_v_2.1 and 2.2 (262, 269). These changes may, in part, reflect the influence of the silent subunit K_v_9.1 in hetero-tetramers with both K_v_2.1 and K_v_2.2 (67, 247, 270, 271). Nerve injury downregulates K_v_9.1 in DRG neurons and this may alter behavior of K_v_9.1~K_v_2.1~K_v_2.2 hetero-tetramers (270). Selective downregulation of the *Kcns* gene in DRG *in vivo* but not in other tissues, reduces K_v_9.1 expression and promotes changes in pain behavior consistent with its role in onset of neuropathic pain (67, 270). This suggests that restoring *Kcns1* activity in the periphery has therapeutic potential in chronic pain (67).

As seen with K_v_2.1, nerve injury downregulates mRNA for K_v_2.2 in DRG (255, 269). Since K_v_2.2 currents are also affected by the presence of K_v_9.1 in hetero-tetramers this give further credibility to potentiation of K_v_9.1 as a therapeutic approach (Table 1).

#### No Role for K_v_3.1 and 3.2 in Neuropathic Pain

Although immunohistochemical, biophysical and Western immunoblot studies have identified these isoforms in DRG (272), there is little or no evidence for injury-induced changes in their expression or function (255).

### Decreased Expression and Therapeutic Modulation of K_v_7.2/7.3 M- Channels

#### Role of K_v_7.2/7.3 in Neuropathic Pain

M-channels are the K_v_7.2 and K_v_7.3 products of the *KCNQ2/3* genes (273). They are activated by depolarization in a similar fashion to delayed rectifiers but do not inactivate over periods of many minutes. This and the fact that M-channels start to activate at normal resting potential means that they play an important role in determining neuronal excitability and accommodation of firing (274, 275). Whole-cell M-current is reduced in a model of bone cancer pain (276), selective knockdown of K_v_7.2 in DRG causes hyperalgesia (277) and peripheral nerve injury induces substantial downregulation of K_v_7.2 protein (239). The observation that the M-channel openers such as flupirtine and retigabine alleviate hyperalgesia in several rodent pain models (239, 278, 279) initiated considerable interest in the potential therapeutic use of this type of drug (280–285).

#### Pharmacological Manipulation of K_v_7.2/7.3

Although a clinical study of retigabine in post herpetic neuralgia failed to meet its efficacy endpoint (68), at least 200 K_v_ activators are currently under development (285). It has also been observed that the natural products, mallotoxin (MTX) and isovaleric acid (IVA), act synergistically to open neuronal KCNQ channels. This combination has been shown to suppress pentylenetetrazole-induced tonic seizures in mice but has not yet been examined in pain models (71). Similar effects were seen with (E)-2-dodecenal (E-2-D), a natural product derived from cilantro leaves (72). It has been suggested that co-administering MTX, IVA or E-2-D with retigabine may be highly effective in opening of KCNQ2/3 channels (71) (Table 1).

A novel K_v_7.2 activator known as SCR 2682 was described recently (69). Acute application of SCR 2682 augments M-currents in DRG neurons and alleviates nerve injury induced pain *in vivo*. Both effects are reversed by M-channel inhibitor XE991. SCR 2682 also increases KCNQ2 *mRNA* and K_v_7.2 protein expression in a rodent model of neuropathic pain (70) but its exact mechanism of action is yet to be determined.

K_v_7 thus retains its potential as a drug target for neuropathic pain (Table 1); chemical modification of the retigabine structure may provide new and effective therapeutic agents.

The effects of nerve injury on expression of *KCNQ* depend on the actions of inflammatory mediators (286) and/or inhibition of transcription by repressor element 1-silencing transcription factor (REST also known as neuron-restrictive silencing factor, NRSF) (239, 287). Overexpression of REST in DRG neurons strongly suppresses M-current density, increases excitability induces mechanical and thermal hyperalgesia (288). Specific knockout of REST in DRG prevents injury-induced downregulation of REST target genes and prevents the development of hyperalgesia in various models of neuropathic pain; an effect that can be restored by REST overexpression (288).

REST inhibits transcription by recruiting the co-repressor complexes SIN3A/B and REST corepressor 1; these complexes modify target gene regions through the action of HDAC1/2, the histone demethylase LSD1 and the histone methyltransferase G9a (289, 290). Inhibition or genetic deletion of G9a in DRG abolishes injury-induced down-regulation of K_v_7.2 and reduces neuropathic hyperalgesia. G9a may have an important role in K^+^ channel regulation as it has also been implicated in injury induced suppression of K_v_1.4, K_v_4.2, and BK channels (K_Ca_1.1) (250). Two small molecule inhibitors of G9a are available, namely BIX01294 and UNC0638, both of which attenuate neuropathic pain in rodent models (80, 81). Although there is considerable interest in developing histone methyltranferase inhibitors in cancer treatment (291), to the best of our knowledge neither BIX01294 nor UNC0638 have been examined for treatment of pain in the clinic. Further development of drugs of this type may lead to new approaches to pain management (Table 1).

### Decreased Expression and Therapeutic Modulation of A-Channels

A-type potassium channels are largely inactivated at the normal resting potential of DRG neurons and this inactivation must be removed by hyperpolarization prior to depolarization to effect channel opening. Once activated, A-channels display profound and usually rapid inactivation. Despite the rather complex protocols required to activate A-currents in a voltage-clamp experiment, A-channels play a role in neuronal activity by modulating the shape of action potential afterhyperpolarizations, participating in action potential repolarization (247, 292) and increasing the latency of depolarization activated action potentials. There are several different types of A-current distinguished by their sensitivity to the channel blocker 4-aminopyridine (4-AP) and by their rate of inactivation. Nerve injury, including diabetic neuropathy decreases whole-cell A-current in DRG neurons (237, 238, 254, 293, 294). This reflects altered functionality of K_v_1.4, K_v_3.4, and K_v_4's, which are the dominant A-current types in DRG (28, 244). A-channels seem especially sensitive to changes induced in models of diabetic neuropathy (254).

#### Role of K_v_1.4 in Neuropathic Pain

mRNA for K_v_1.4 is downregulated in several models of neuropathic pain, including a model of diabetic neuropathy (238, 250, 254, 255). Knockdown of K_v_1.4 with siRNA causes allodynia (295) and miR-17-92 overexpression downregulates A-channels and promotes hyperalgesia (296). The molecular mechanism of altered K_v_1.4 expression is similar to that for K_v_7.2 described above (250). This means that the effectiveness of G9a inhibitors in inhibiting neuropathic pain (80, 81) may involve preservation of function of both K_v_7.2 and K_v_1.4 after injury (Table 1).

#### Role of K_v_3.4 in Neuropathic Pain

Kv3.4 are high threshold A-channels that are particularly sensitive to 4-AP block. Nerve injury decreases expression of K_v_3.4 immunoreactivity (297) and mRNA is reduced in a model of diabetic neuropathy (254). K_v_3.4 antisense produces mechanical hypersensitivity (297). It has also been reported that injury to the spinal cord *per se* causes K_v_3.4 dysfunction in DRG (298). This may reflect the action of excitatory mediators released from the spinal site of injury. This raises the possibility that therapeutic control of DRG function may not only be beneficial for peripheral neuropathy, it may also have benefit for managing pain originating from spinal cord injury.

#### Role of K_v_4.1, 4.2, and 4.3 in Neuropathic Pain

Immunoreactivity and/or mRNA for all three K_v_4 channels is found in DRG neurons (28, 255, 294, 297, 299) with differences in their distribution across different neuronal types (300, 301). Decreased function of all K_v_4 channels occurs after peripheral nerve injury (75, 244, 254, 293, 297, 299, 302), and knockdown of K_v_4.1 and its modulatory subunits or antisense to K_v_4.3 causes mechanical hypersensitivity (297, 299). Taken together these observations strongly suggest malfunction of K_v_4 channels in neuropathic pain.

The expression and function of K_v_4 channels in DRG is controlled by signaling pathways such as MAPK (293), K_v_4 channel interacting proteins (KChIPs) and dipeptidyl-peptidase-like proteins (DPPLs) (303–305). The aforementioned neuron restrictor silencer factor (REST), which controls expression K_v_7.2, also effects suppression of transcription of the K_v_4.3 gene (*KCND3*) after nerve injury (302).

#### Pharmacological Manipulation of K_v_4

Since no activators of K_v_4 channels are available, targeting accessory subunits of A-channels may provide an alternative strategy (244). DPPLs and KChIPs not only govern the biophysical properties of K_v_ channels. They also impact channel assembly, channel trafficking to and from the cellular surface, and targeting of channels to different cellular compartments (304). The compound NS5806 has been reported to potentiate K_v_4 currents in a KChip dependent manner (73, 74) and has recently been shown to attenuate cold allodynia in a rodent model of trigeminal neuralgia [(75), Table 1].

### Decreased Expression and Therapeutic Modulation of Ca^2+^-Sensitive K^+^ Channels

Ca^2+^-sensitive K^+^ channels fall into three broad categories; K_Ca_1.1, also known as BK or maxi g_K, Ca_ channels which are high conductance, voltage-sensitive and blocked by low concentrations of tetraethylammonium; K_Ca_2.1,2.2 and 2.3 which are apamin sensitive, low conductance, and voltage-independent and K_Ca_3.1 which are intermediate conductance and clortrimazole sensitive (246). In neurons, these channels play a major role in the determination of spike width, repolarization, after hyperpolarization amplitude and duration, repetitive discharge characteristics, accommodation and overall excitability. As with other K^+^ channel types, their potential as therapeutic targets is limited by their ubiquitous distribution and function in both excitable and non-excitable tissues (246).

#### Role of K_Ca_1.1/BK Channels in Neuropathic Pain

BK channels are encoded by the *KCNMA1* gene and are present in all DRG neurons (240, 306–308). Their functional expression is reduced by peripheral nerve injury (236, 240, 309). This is associated with decreased expression of *KCNMA1* and channel protein (250, 310). Their involvement in generation of pain is suggested by the observation that overexpression of BK increases mechanical threshold in a rodent neuropathic pain model (311). Also, the K_Ca_1.1. blocker, iberiotoxin reduces mechanical withdrawal threshold.

#### Pharmacological Manipulation of K_Ca_**1.1/BK Channels**

Intrathecal injection of the K_Ca_1.1 channel opener [1,3-dihydro-1-[2-hydroxy-5-(trifluoromethyl)phenyl]-5-(trifluoromethyl)-2H-benzi midazol-2-one] dose-dependently reverses allodynia and hyperalgesia in nerve-injured rats but had no significant effect on nociception in control rats (310). This substance is one of several BK activators available including the highly effective GoSlo-SR family of anthraquinone analogs (312). Others include NS1619 (313, 314), NS11021 (315, 316), NS13558 (317), and 12,14-dichlorodehydroabietic acid (diCl-DHAA) (318). Because these drugs have profound effects on tissues such as cardiac myocytes and certain smooth muscles, they are unlikely to be of practical use in pain management.

On the other hand, there is considerable discussion in the literature relating to the efficacy of cannabinoids in neuropathic pain (102, 106, 107) and it has been suggested that augmentation of BK function may contribute to their potential therapeutic effect (103).

As was described for K_v_7.2 and K_v_1.4, injury-induced downregulation of *KCNMA1* in DRG is a result of G9a activation (250). This underlines the potential therapeutic application of G9a blockers such as BIX01294 and UNC0638 (Table 1).

#### Role of K_Ca_2.1, 2.2, 2.3, and 3.1 in Neuropathic Pain and K_Ca_3.1 as a Therapeutic Target

There is little information about the possible role of K_Ca_2 channels in pain but several recent reports have drawn attention to the possible role of K_Ca_3.1 (Table 1). Although K_Ca_3.1 knockout-mice show increased sensitivity to noxious chemical stimuli they exhibit normal behavioral responses to acute nociceptive, persistent inflammatory, and persistent neuropathic pain (319). Despite this, the K_Ca_3.1 channel opener, ASP0819, modulates nociceptive processing and *in vivo* action potential activity in peripheral nerves in an animal model of fibromyalgia (320) and preliminary investigation of its action in the clinic have provided evidence of efficacy with minimal side effects (321).

### Decreased Expression and Therapeutic Modulation of Inwardly Rectifying K^+^ Channels

Although a variety of two transmembrane domain inwardly-rectifying K^+^ channels are found in DRG neurons (28), by far the most information of relevance to pain mechanism and potential management relates to findings on the K_ATP_ channel; K_ir_6.2 (243, 322, 323).

#### Role of K_ir_6.2/K_ATP_ Channels in Neuropathic Pain

K_ATP_ channels play an indispensable role in pancreatic insulin secretion as a result of their inhibition by intracellular ATP and their activation by ADP (248). Sulphonylurea receptors (SUR or ATP binding cassettes) co-assemble with channel proteins (324). K_ATP_ channel activation can be achieved by the anti-hypertensive agents, diazoxide and pinacidil and their anti-nociceptive actions have been recognized for many years (325). Nerve injury reduces K_ATP_ currents and channel activity in DRG neurons (323, 326) and although there are several reports of the efficacy of K_ATP_ openers in neuropathic pain models (76–79), these findings do not appear to have been exploited in the clinic.

### Decreased Expression and Therapeutic Modulation Tandem Pore Domain K^+^ Channels

#### Downregulation of TRESK, TASK3, and TWIK1 by Nerve Injury and Relevance to Neuropathic Pain

Four transmembrane-domain tandem pore domain (K_2p_) channels account for K^+^ leak conductance and set the resting membrane potential of most excitable cells including DRG neurons (28, 249, 327). TRESK (k_2p_18) channels seem particularly important in this regard (328). Their potential relevance to neuropathic pain is supported by the observation that sciatic nerve transection reduces TRESK/(k_2p_18)/*KCNK18* mRNA to a greater extent than other K_2p_ channels in DRG and *in vivo* knock down decreases threshold to painful mechanical stimuli (329, 330). Other K_2P_ channels such TASK3 (K_2p_9) and TWIK1 (K_2P_1) are also down-regulated by spared nerve injury (SNI) (331).

#### Therapeutic Modulation of Tandem Pore Domain K^+^ Channels

Although activation of K_2_P channels contributes to the therapeutic effectiveness of volatile anesthetics such as isoflurane (327, 332) it is obviously impractical to use these drugs for long term pain management. The novel TREK2/K_2p_10.1 activator GI-530159 decreases DRG excitability (333), but its possible effectiveness in pain models has not yet been reported.

## Voltage-Gated Ca^2+^ Channels

Voltage-gated Ca^2+^ channels (VGCCs) have been studied for more than 20 years as potential therapeutic targets for chronic pain (93, 334, 335). They are subdivided into high-voltage activated (HVA) L-types (Ca_v_1.1, Ca_v_1.2, Ca_v_1.3, and Ca_v_1.4), P/Q-type (Ca_v_2.1), N-type (Ca_v_2.2), and R-type (Ca_v_2.3) and low voltage activated (LVA) T-types (Ca_v_3.1, Ca_v_3.2, Ca_v_3.3) (93, 336, 337). The distribution of channels in DRG muscle afferents is Ca_v_2.2 (N-type) > Ca_v_2.1 (P/Q-type) > Ca_v_1.2 (L-type) (338). There is little or no evidence for the expression of Ca_v_1.1, Ca_v_1.3, and Ca_v_1.4 in DRG as these are found mainly in heart, skeletal muscle, endocrine cells, smooth muscle and the vestibular system (93, 336). R-type Ca_v_2.3 and P/Q type Ca_v_2.1 also appear to be absent from DRG (338).

VGCC set DRG neuron excitability either by generating voltage-gated inward currents or by producing outward currents following the activation of Ca^2+^ sensitive K^+^ channels (236). Influx of Ca^2+^ through HVA channels triggers release of excitatory neurotransmitters from presynaptic vesicles and thereby determines dorsal horn excitability. The role of VGCC in neuropathic pain and pain therapeutics in general is well-established (24, 93, 236, 339–344). This is underlined by the therapeutic effectiveness of the N-type Ca^2+^ channel blocker ziconotide (339), the established use of gabapentinoids which bind to the α2δ-1 regulatory subunit of HVA Ca^2+^ channels (3, 345, 346) and the observation that N-type VGCC knockout mice exhibit reduced signs of both inflammatory and neuropathic pain (347). The α2δ-1 subunit plays a major role in the expression and function of VGCC (346, 348) and α2δ-1 gene deletion delays mechanical hypersensitivity in response to peripheral nerve damage (349).

Since VGCC are responsible for triggering release of neurotransmitter, blocking, or genetically deleting these channels in peripheral neurons reduces synaptic input to the spinal cord (93) and ω-conotoxin GVIA reduces synaptic potentials in the spinal cord (350).

Early experimental investigations of the effects of nerve injury on VGCC function were completed some years before the establishment of formal structural and genetic definitions of channel subtypes. Axotomy or chronic constriction injury reduced function of HVA channels in the cell bodies of DRG neurons (236, 342, 351) and there was no preferential loss of N-type vs. L-type channels (236). As with Na^+^ and K^+^ channels, the structural and genetic definition of VGCC subtypes (336) has refined descriptions of injury induced changes and enabled the logical development of current and potential therapeutic agents (93, 335, 339).

### Therapeutic Modulation of HVA Ca^2+^ Channels

#### L-Type Ca_v_1.2 Channels in Neuropathic Pain

Although these L-type VGCC are present in rodent DRG (338), gain of function mutations in humans do not express a pain phenotype (93). On the other hand, following CCI of the sciatic nerve, the “classical” dihydropyridine, nitrendipine reduces the frequency of spontaneous EPSC's in rat lamina II (*substantia gelatinosa*) neurons. It also, albeit rather weakly, attenuates mechanical allodynia. These effects have been attributed to injury-induced upregulation of α2δ-1 and increased expression of Ca_v_1.2 after nerve injury (348). Anti-Ca_v_1.2 siRNA or selective knockdown of Ca_v_1.2 in the spinal dorsal horn but not in DRG has been shown to reverse the nerve injury associated mechanical hypersensitivity of dorsal horn neurons. This implies that postsynaptic effects such as CREB phosphorylation in the spinal dorsal horn may also contribute to the participation of Ca_v_1.2 in neuropathic pain (352, 353). It may relate to the finding that α2δ-1 remodels Ca_v_1.2 voltage sensors and allows Ca^2+^ influx at physiological resting potentials (354).

#### Pharmacological Manipulation of L-Type Ca_v_1.2 Channels

Since we could only find one very old report of clinical effectiveness of classical dihydropyridine, nifedipine in complex regional pain syndrome (355), it is presently assumed that L-type Ca^2+^ channels play a far smaller role in the etiology of neuropathic pain than N- or T-types (see below). This position may however need revision in the light of recent descriptions of prevalent nifedipine sensitive channels in human DRG neurons (356).

Some novel benzodiazepines exhibit selective T-channel block (357) whereas others block both Ca_v_1.2 L-type and Ca_v_3.2 T-type calcium channels (358). To the best of knowledge there are no reports of the effectiveness of these agents in the clinic.

#### Role of Ca_v_2 Channels in Neuropathic Pain

Ca_v_2 channels are the main subtype found in primary afferent terminals (93, 359). Ca_v_2.1 (N-type) and Ca_v_2.2 (P/Q type) both contain a synaptic protein interaction site (synprint) that interacts with SNARE proteins (syntaxin and SNAP-25) (360, 361). By this mechanism, channels can be closely associated with synaptic vesicles that govern release of neurotransmitter from primary afferent terminals. Although suppression of N-type Ca^2+^ channel current increases the excitability of DRG cell bodies by concomitant decrease of BK function (236, 306), this effect is overridden *in vivo* by the actions of Ca_v_2 blockers to prevent neurotransmitter release from primary afferent terminals.

#### Pharmacological Manipulation of Ca_v_2 Channels

As already mentioned, the Ca_v_2.2 blocker ziconotide which is a synthetic version of ω-conotoxin MVIIA from the cone snail *Conus magnus* (362) is employed in pain management. The main drawback is that it needs to be delivered directly to the spinal cord *via* an intrathecal drug delivery system. Zicononotide (Prialt) is usually only effective in patients with severe, intractable forms of chronic pain such as that associated with cancer (82, 363).

There is therefore considerable interest in developing small molecule blockers of Ca_v_2 channels that may be effective orally or perhaps by intravenous injection (24, 93, 339, 344). In our previous review (3) we drew attention to the state-dependent Ca_v_2 blockers ZC88 (83, 84), A-1264087 (85, 364), and TROX-1 (86, 87, 365). Although all of these drugs display anti-allodynic efficacy in rodent models of neuropathic pain (344), there is as yet no evidence of any clinical efficacy.

Two tetrahydroisoquinoline derivatives have also been shown to display effectiveness in animal models (366, 367) but again clinical efficacy has not yet been demonstrated.

A permanently charged cationic derivative of an N-type calcium channel-blocker was recently synthesized (97). These authors anticipated that this charged compound (known as CNCB-2) would only be effective when applied intracellularly by a mechanism analogous to QX-314 block of Na^+^ channels (60). Surprisingly, extracellular application of CNCB-2 was more effective than intracellular application in inhibiting Ca_v_2.2 channels. Inhibition was achieved without channel opening. Moreover, and quite unexpectedly, the compound was also highly effective in inhibiting Na_v_1.7 when applied extracellularly. CNCB-2 reduced excitability of mouse DRG neurons and produced long lasting analgesia in several pain models. Given the seminal role of Na_v_1.7 in the etiology of many forms of neuropathic pain (8, 114), bifunctional compounds such as CNCB-2, show considerable promise as therapeutic agents (Table 1).

Ca_v_2.2 interacts with collapsin response mediator protein 2 (CRMP2) which directs the channels to presynaptic terminals (368). Interestingly it has been reported that impairment of CRMP2 function using a homopolyarginine (R9)-conjugated CBD3-A6K peptide inhibits Ca_v_2.2-CRMP2 interaction, diminishes surface expression of Ca_v_2.2 and alleviates tactile allodynia and ongoing pain in a rodent model (369). This observation suggests that CRMP2 may be developed as a novel therapeutic target.

N-type Ca^2+^ channels are modulated by G_i/o_ coupled agonists (157, 370). The α_2_-adrenoceptor agonist, clonidine displays anti-allodynic actions in a rodent model (371) and meta-analysis of clinical trials reveals clinical efficacy (372). Effects of clonidine may be mediated by α_2_-adrenergic inhibition of neurotransmitter release leading to modulation of pain processing at the spinal level (5) and/or by attenuation of aberrant interactions between sympathetic and sensory nerves in the periphery (156, 157, 373). Its effectiveness is however limited to subsets of patients within the diabetic neuropathy, complex regional pain syndrome or postherpetic neuralgia cohorts (88–92). In view of the restricted effectiveness of clonidine, it does not meet the criteria for first line treatment of neuropathic pain (1) (Table 1).

Gabapentinoids on the other hand are relatively but not completely effective in a variety of manifestations of neuropathic pain; about 31% of patients see clear benefit (96). Their mechanism is still incompletely understood but clearly involves impediment of transport of Ca_v_2 channels to nerve terminals and their uncoupling from the neurotransmitter release process following interaction with their α2δ-1 accessory subunits (3, 94, 374). This occurs in both primary afferents and dorsal horn (95). Apart from the introduction of pregabalin (375) and an enacarbil derivative of gabapentin with improved oral bioavailability (376), there have been no major developments in the pharmacology of α2δ-1 ligands since their introduction in the 1990's. Since gabapentinoids act intracellularly, we have suggested that their effectiveness may be increased by allowing them to enter neurons *via* the open pore of TRPV1 channels (377).

Since Ca_v_2.2 channels are found in pancreatic β-cells and are involved in the secretion of insulin (378) it remains to be established whether Ca_v_2.2 blockers have undesirable effects on blood glucose levels. On the other hand, Ca_v_2.2 has been implicated in microglial function (379, 380). This raises the possibility that some of the beneficial effects of Ca_v_2.2 blockers result from actions on microglia.

### Therapeutic Modulation of LVA Ca^2+^ Channels (T-Channels)

#### Role of Ca_v_3.2 in Neuropathic Pain

T-type, LVA, Ca^2+^ channels (Ca_v_3.1, Ca_v_3.2, Ca_v_3.3) play important roles in setting neuronal excitability (93, 336, 381) and in transmitter release from primary afferent terminals (382, 383). As with Ca_v_2 channels, this later function may involve interaction of Ca_v_3 channels with the synaptic vesicle release proteins syntaxin 1A and SNAP25 (synprint) (384). DRG neurons express Ca_v_3.2 and 3.3 but not 3.1 (385–387). T-type calcium currents are increased in rodent DRG neurons after peripheral nerve injury in a model of diabetic neuropathy and after injury to the spinal cord *per se* (383, 388–390).

Although there are no reported mutations of Ca_v_3.2 that produce a painful phenotype in humans, most of the work relevant to pain mechanisms has involved this channel as opposed to Ca_v_3.1 or Ca_v_3.3 (93, 334, 335, 339, 391–393). Ca_v_3.2 is expressed in low-threshold mechanoreceptors and conditional knockout of the channel in this neuronal subtype has implicated Ca_v_3.2 in allodynia linked to neuropathic pain (394). Several mechanisms control the functional expression of Ca_v_3.2 channels.

(i) Upregulation of the deubiquitinase, USP5 by the action of the inflammatory mediator interleukin-1β. This impairs Ca_v_3.2 ubiquitination thereby protecting it from proteasomal degradation and prolonging its surface expression (383, 395, 396). Knockdown of USP5 *in vitro* increases Ca_v_3.2 ubiquitination and reduces Ca_v_3.2 whole-cell currents and since impairment of USP5 function *in vivo* attenuates mechanical hypersensitivity in both inflammatory and neuropathic mouse models, this enzyme may represent a future therapeutic target (335, 383). Progress in this direction involves the observations that Ca_v_3.2/USP5 interactions are interrupted by the anti-parasitic agent, suramin and by a TAT-cUBP1-USP5 peptide and both substances show analgesic activity in neuropathic and inflammatory pain models (99, 100) (Table 1).

(ii) Glycosylation and enhancement of channel trafficking in diabetic pain (397, 398). Deglycosylation of Ca_v_3.2 with neuramidase reverses hyperalgesia in a model of diabetic neuropathy (398) (Table 1).

(iii) BDNF stimulation of TrkB coupled to PI3K-p38-PKA signaling in trigeminal neurons (399). Although a range of small molecule TrkB inhibitors are available (400), the multiple biological actions of BDNF in the developing and mature nervous system, preclude the use of these agents in pain management (401).

(iv) Ca_v_3.2 channels interact with the scaffold protein Rack-1 [receptor for activated C kinase 1 (402)]. Whole-cell Ca_v_3.2 current and channel expression in the plasma membrane is reduced when Ca_v_3.2 and Rack-1 are co-expressed in tsA-201 cells. Molecular interaction between the two proteins was demonstrated by co-immunoprecipitation. These findings assume special significance in the light of the suggested role for Rack-1 in neuropathic pain (403).

#### Pharmacological Manipulation of Ca_v_3.2

Although T-type Ca^2+^ channel blockers such as the anticonvulsant ethosuximide increases withdrawal thresholds in nerve-injured rats (404), clinical studies of its effectiveness in pain management have been disappointing (98). A similar picture emerges for other small molecule blockers of Ca_v_3.2, most of which showed considerable promise in preclinical studies yet failed to exert significant effects in cohorts of pain patients (334).

For example, ABT-639 showed promise in preclinical studies (405–407) but clinical results have been disappointing (86, 334); it did not treat pain in patients with diabetic neuropathy (408) and has now been discontinued.

Also, because TTA-P2 is a highly selective Ca_v_3.2 channel blocker that has minimal effects on other cation channels, it is used extensively in laboratory investigations of T-channel function. Although it is effective in rodent models of chronic inflammatory pain and diabetic neuropathy (409) we could find no reports of its efficacy in the clinic.

Similarly, TTA-A2 is used extensively in laboratory investigations (395) as it has higher affinity for Ca_v_3.2 than Ca_v_31.1 (410). Although it is effective in rodent models of irritable bowel syndrome (410), no clinical studies appear to have been done.

Z944 is another high-affinity T-type channel blocker that is effective against Ca_v_3.1, Ca_v_3.2, and Ca_v_3.3 with little affinity for other Ca^2+^ channel types (411). Its effectiveness in murine pain models may reflect it actions on spinal and thalamic neurons (412, 413). So far, the results of phase 1 and phase 2 trials appear promising (101) (Table 1). Although there does not seem to be any preclinical information regarding the effectiveness of the N-(1-benzyl-1H-pyrazol-3-yl)-2-phenylacetamide derivative ACT-709478 in animal models of neuropathic pain (414), it appears to be showing promise in phase 2 trials (101) (Table 1).

As already mentioned the has been shown to also selectively block Ca_v_3.2 (49).

Cannabinoids, which are effective in some neuropathic pain cases (108, 109), inhibit recombinant human T-type (Ca_v_ 3.1, 3.2) Ca^2+^ channels (104) and as mentioned above, augment BK (K_Ca_1.1) currents. Intrathecal injection of the CB1/CB2 receptor agonist NMP-7 inhibits injury-induced neuropathic pain in a rodent model. This effect involves CB2 receptors and Ca_v_3.2 channels (415). To the best of our knowledge, NMP-7 has not yet progressed to clinical trials but its preclinical effectiveness led to the development of the derivative [N-((1-(2-(tertbutylamino)-2-oxoethyl)piperidin-4-yl)methyl)-9-pentyl-9Hcarbazole-3-carboxamide] (Compound 9) which displays remarkable effectiveness in murine models of inflammatory and neuropathic pain (105).

## HCN-Channels

### Role of HCN2 and 3 in Neuropathic Pain

There are 4 isoforms of hyperpolarization-activated cyclic nucleotide–gated (HCN) channels (416); HCN1, HCN2, HCN3, and HCN4 coded by *HCN1, HCN2, HCN3*, and *HCN4* genes. HCN3 are distinguished by their relatively low sensitivity to intracellular cAMP (416). HCN channels underlie neuronal H-current (I_h_).

I_h_ is upregulated in DRG after nerve injury (417) where it drives spontaneous activity (30, 418–421) and increases transmitter release from primary afferents (422, 423).

Whereas, HCN1 and HCN4 channels are primarily expressed in cardiac pacemakers, HCN2 channels are mainly expressed in neurons. They have emerged as a promising peripheral drug target for neuropathic as well as inflammatory pain (3, 27, 335, 418–420, 424–426).

HCN2 is expressed is expressed in about 50% of small somatosensory neurons, which are mainly nociceptors. It plays an important role in the control of firing frequency in response to noxious stimuli (420). Indeed deletion of HCN2 in nociceptive neurons prevents the development of inflammatory and neuropathic pain (420).

HCN3 is expressed in most DRG neurons and is persistently activated at their normal resting potential thereby contributing to membrane resistance. Neurons from HCN3-knockout mice exhibit increased input resistance and increased excitability, but experience similar levels of mechanical allodynia and thermal hyperalgesia to wild-types following nerve injury. This suggests that HCN3 plays little or no role in processing of neuropathic pain (427).

### Pharmacological Manipulation of HCN Channels

Ivabradine which blocks HCN1, 2, and 4 (416) is used clinically to treat chronic angina and heart failure (335). It abrogates signs of neuropathic pain in animal models through peripheral action on small sensory neurons (418, 425). The effectiveness of ivabradine may be in part attributed to its ability to increase K_v_7 channel activity (428) and perhaps actions at the thalamic level as seen with the classical I_h_ blocker ZD7288 (429). Although we found ivabradine administered to nerve injured rats at a dose that significantly reduced mechanical allodynia was without noticeable effect on arterial pressure and produced only a 15% reduction in heart rate, its cardiovascular actions have detracted from its use as an analgesic agent in the clinic (430).

More recent work has thus focused on the search for selective HCN2 blockers (431) that may abrogate hyperexcitability of DRG neurons without affecting the HCN1 channels that are responsible for controlling cardiac rhythmicity (27). However, to the best of our knowledge, no small molecule blockers are as yet available.

## Discussion

Unlike morphine for nociceptive pain, there is no equivalent panacea for neuropathic pain. The well-tried therapeutic approaches to neuropathic pain (gabapentinoids, tricyclic antidepressants and serotonin-noradrenaline reuptake inhibitors) retain their position in the “winners circle” of effective agents (1, 2). They have not yet been superseded by any of the treatments or approaches listed herein (223). Although a variety of therapeutic approaches have been mentioned above, Table 1 lists only those compounds that show considerable promise as therapeutic agents.

In the final section of the review, we suggest future considerations and refinements that may enable the further development and usage of peripherally-acting drugs as possible therapeutic approaches to pain management.

### Use and Structural Refinement of Promising Candidate Molecules

Many drugs that are effective in animal models fail to lead to useful clinical agents because of dose limiting toxicities, unfavorable pharmacokinetics or “off target effects.” Some of these issues can be minimized by chemical modification of safe pharmacological agents or drug repurposing.

#### Therapeutic Potential of Na^+^ Channel Blockers

Several Na^+^ channel blockers show promise as therapeutic agents or as lead compounds for structural refinements (Table 1).

The first is the Na_v_1.7 blocker, vixotrigine (CNV1014802, BIIB074, or GSK-1014802) (184, 185). The outcomes of a phase III clinical trial for effectiveness in trigeminal neuralgia (NCT03637387) and phase II trial for small fiber neuropathy are eagerly awaited (47).

The Na_v_1.7 blocker PF-05089771 failed to meet defined efficacy criteria in patients with painful diabetic peripheral neuropathy (46). Since its use in clinical trials would have been contingent on establishment of safety for use in humans, it may serve as a safe lead compound for the development of more effective agents.

Certain natural toxins, notably those from various types of tarantula venom show selectivity and high affinity for Na_v_1.7 as well as analgesic effects in various pain models. One of the most promising agents is Tap1a as this interacts with both Na_v_1.7 and the T-type Ca^2+^ channel, Ca_v_3.2 (49, 50). Recent studies of Tap1a have shown that it interacts with voltage-sensor domain II of Na_v_ channels with nanomolar affinity. Structural modification of Tap1a has produced two peptides Tap1a-OPT1 and Tap1a-OPT2 that exhibit increased affinity for Na_v_1.1, Na_v_1.2, Na_v_1.3, Na_v_1.6, and Na_v_1.7. Intraplantar injection of Tap1a-OPT1 reduces Na_v_1.7/OD1-induced spontaneous pain behaviors in a murine model. Moreover the anti-nociceptive effect of Tap1a-OPT1 is significantly greater than the native peptide (50).

Although the selective Na_v_1.8 blockers A803467 and PF-01247324 attenuate allodynia in a rodent model (221, 222), they have not yet been examined in the clinic (223). The pro-drug VX-150 is metabolized into a highly selective Na_v_1.8 blocker which exhibits analgesic activity in healthy volunteers (57). Expression of Na_v_1.8 in peptidergic DRG neurons is controlled by NGF (215) and the NGF binding antibody tanezumab is effective in various pain states (58). In fact, its safety and efficacy in humans identifies tanezumab as one of more the promising new drug candidates for chronic and neuropathic pain (see Clinical Trials Government Identifiers: NCT02528188 and NCT02528188). Small molecule peripherally acting TrkA inhibitors have recently been described (432, 433).

We have also described the idea of combining cationic local anesthetics with TRPV 1 activators (60, 61, 233), although this seems to work well in animal models, this approach has not yet been demonstrated in a clinical situation.

#### Therapeutic Potential of K^+^ Channel Activators

Although a clinical study of retigabine in post herpetic neuralgia failed to meet its efficacy endpoint (68), there is considerable interest in its structure as template for ligand-based drug design of K_v_7.2/3 activators (434); at least 200 K_v_ activators are currently under development (285). Certain natural products augment K_v_ currents and it has been suggested that these might augment retigabine effectiveness (71).

The sulphonylurea compound NS5806 which augments K_v_4.3 type A-currents in animal models (75) is yet to be examined in the clinic.

A phase 2a clinical trial of the K_Ca_3.1 channel opener, ASP0819 for fibromyalgia (NCT03056690), has provided evidence of efficacy with minimal side effects (321). As mentioned above, little, or no success has been realized with other direct activators of K_v_1, 2, 3 or 4 or K_Ca_1 or 2.

#### Therapeutic Potential of Ca^2+^ Channel Blockers

N-type Ca_v_2 channels have been recognized as targets for anti-allodynic drugs for many years. The limitations to the use of the channel blocker ziconotide and the α2-adrenoceptor ligand clonidine have already been alluded to (363). Although gabapentinoids interact indirectly with Ca_v_2 *via* their α2δ-1 subunits they are neither universally effective or without undesirable adverse effects (96). As mentioned above, a few small molecule Ca_v_2 blockers are in development but none have as yet been tested in a clinical situation. The compound CNCB-2 is of special interest as it blocks both Ca_v_2.2 and Na_v_1.7 channels (97).

The potential role of Ca_v_3 in neuropathic pain was established about 12 years ago (389, 413, 435) but the classical Ca_v_3 blocker ethosuximide displays only limited effectiveness in the clinic (98). In the interim, several small molecule blockers have appeared such as TTA-P2 and TTA-A2 which are highly selective for Ca_v_3.2. Clinical studies are yet to be initiated or reported. By contrast, phase 1 and 2 clinical studies with two compounds Z944 and ACT-70948 have yielded promising results (101). Interest in cannabinoid modulation of Cav3.1 and 3.2 has led to development of a series of small molecule channel blockers such as “compound 9,” although it is remarkably effective in preclinical studies clinical studies are yet to be initiated (105).

### Improve Assessment of “Pain” as Opposed to Nociception in Rodent Models

Preclinical effectiveness of therapeutic intervention in neuropathic pain is often assessed by examination of drugs' ability to attenuate behavioral indices of pain induced by surgical or chemical lesions to peripheral nerves of experimental animals (436, 437). Typical measurements involve examination of mechanical or thermal withdrawal thresholds or presence of hyperalgesia and or touch or cold-induced pain (mechanical or thermal allodynia). It may be argued however that withdrawal of a foot or limb in response to a noxious stimulus may simply reflect activation of a spinal reflex (438). The inability to measure “pain” *per se* with both its nociceptive and emotional comments may underlie the limited ability of rodent models to predict clinical efficacy (68, 171, 439). In an attempt to assess true pain and its attenuation in rodent models, more recent non-invasive models for assessment of chronic pain involve quantification of indices such as facial grimace score as well as observation of social interaction and nest-building (Turner et al., 2019; Sotocinal et al., 2011) (437). This is complemented by the use operant models such as conditioned place preference protocols. In one version of this, rodents are required to make a conscious choice between being in a pain-inducing environment and an otherwise undesirable environment such as a brightly illuminated space (3, 440–442). The time spent in the undesirable brightly illuminated environment gives an index of the pain the animal is experiencing.

Translation between animal observations and development of effective human therapeutics may thus be improved by the use of these operant and non-invasive protocols.

### Think About Sex

Women are more prone than men to develop neuropathic pain (12, 443–446). A recent genome wide association study revealed that 123 single nucleotide polymorphisms (SNP) at five independent loci were significantly associated with chronic pain in men whereas in women, 286 genome-wide SNPs were found at 10 independent loci (447). Gene-level analyses revealed sex-specific associations with chronic pain with 31 genes associated in females, 37 genes associated in males, and a single gene, DCC, which codes for the netrin 1 receptor associated in both sexes. Interestingly, all 37 chronic pain associated genes in men and 30/31 genes in women were found to be expressed in DRG (447). These findings match the documented, robust differences that exist in the genetic, molecular, cellular and systems-level mechanisms of acute and chronic pain processing that occur in male vs. female rodents and humans (12, 444, 446, 448–450). This means that preclinical studies previously done exclusively on male rodents need to be repeated in females. This is especially the case in the pain field because sexual convergence onto shared behavioral endpoints, such as allodynia or pain sensitivity, may also mask sex differences in underlying molecular and cellular mechanisms (448).

Among the cellular mechanisms so far identified, it has been reported that spinal microglia activation is required for injury-induced hypersensitivity in males whereas activation and invasion of adaptive immune cells such as T-lymphocytes is required in females (451, 452). Macrophage invasion of DRG is predominant in males and not in females (453) and nociception is regulated by spinal serotonin and noradrenaline in male but not in female mice (454). It has also recently been shown that *ex vivo* treatment of live human organ donor spinal cord tissue with BDNF downregulates markers of inhibition and upregulates markers of facilitated excitation in dorsal horn neurons from males but not females (455). Lastly, administration of IL-23 (Interleukin 23) produces mechanical allodynia in female but not male mice and chemotherapy-induced mechanical pain is selectively impaired in female mice lacking IL-23 or its cognate receptor. (456). These authors have suggested that the difference in response may be attributed to the function of sex hormones as IL-23-induced pain is suppressed by androgen and promoted by estrogen.

In the peripheral nervous system, blockade of Na_v_1.8 channels with A-803467 or Ca_v_2.3 with SNX-482 is more effective in females than in males in various models of neuropathic pain (457, 458).

The realization that different mechanisms are engaged to generate pain in males vs. females has obvious therapeutic implications. If spinal serotonin and noradrenaline attenuate pain in male rather than female rodents (454), might SNRI's such as duloxetine and venlafaxine work better in men than in women? As already mentioned the Na_v_1.8 channel blocker A-803467 works better in woman than in men (457). The importance of the incorporation of sex as a variable in future studies cannot be over emphasized (447, 459).

### Recognize Differences in Pain Etiology (Quantitative Sensory Testing and the Personalized Medicine Approach)

Patients with neuropathic pain are heterogeneous in pathophysiology, etiology and clinical presentation (460). Neuropathic pain can result from sources as varied as nerve compression, channelopathy, autoimmune disease, infection, disease or chemotherapy-induced neuropathy and the response of each individual is determined by a multiplicity of factors such as inherited genetic variants, sex, neonatal injury or maternal separation, age, ethnicity, intestinal microbiome, personality variables, and environmental factors (444, 461–467).

There are numerous examples of cellular mechanisms that may contribute to these differences. For example, adult pain responses are primed by neonatal pain experience and this is maintained by central neuroimmune activity (463). A-channels and HCN channels may be especially affected in diabetic neuropathy (26, 254); might A-current activators and HCN blockers be especially useful in this situation? Na_v_1.7 is found in both sensory and sympathetic nerve fibers, might Na_v_1.7 blockers be especially useful in complex regional pain syndromes? By contrast, in animal studies Na_v_1.7 does not appear to be involved in oxaliplatin-induced painful neuropathy (123) yet does appear to be involved in that seen with paclitaxel (154). Does this mean that Na_v_1.7 blockers might only be effective in subgroups of patients with chemotherapy induced neuropathy (CIPN)?

Perturbations of Na_v_1.6 function may contribute to trigeminal neuralgia (141), might Na_v_1.6 blockers be of special value in this situation? Beyond the peripheral nervous system, the neuronal subtypes in the dorsal horn that are involved in generation of mechanical allodynia is defined by the nature of peripheral nerve injury (468). This likely relates to the observation that CCI of the sciatic nerve produces transient allodynia in animal models whereas that produced by SNI is persistent (469, 470).

In the clinic, various subtypes of neuropathic pain may be identified using quantitative sensory testing (QST). This involves formalization and quantification of an existing battery of neurological tests, such as response to von Frey filaments, vibration, heat, pressure and cold as well as dynamic allodynia and wind-up ratio (460, 471). Findings are compared with large datasets that represent normal responses to sensory tests. Neuropathic pain patients can then be grouped into clusters based on their sensory profiles and that this may have a role in determining treatment (472, 473). Three distinct subgroups with characteristic sensory profiles have already been identified in patients with peripheral neuropathic pain (460). Cluster 1 showed a loss of small and large fiber function in combination with paradoxical heat sensations. Cluster 2 was characterized by preserved sensory functions in combination with heat and cold hyperalgesia and mild dynamic mechanical allodynia and Cluster 3 was characterized by a loss of small fiber function in combination with pinprick hyperalgesia and dynamic mechanical allodynia. The validity of QST is supported by the observation that *post-hoc* analysis of responders to treatments in clinical trials suggest that clinical effectiveness may cluster according to pain phenotype (472).

In view of this, can signs and symptoms observed in each individual patient in the clinic be traced back to underlying pathophysiology? This would permit a “personalized medicine approach” that would dictate the most appropriate therapeutic approach (437, 474, 475). Such an approach may necessitate better “harmonization” between preclinical studies and clinical observations. Thus, while studying chemotherapy-induced pain in rodents may be an appropriate model for understanding CIPN in the clinic, it is less clear how classical rodent pain models such as SNI or CCI relate to the multiplicity of chronic pain presentations in the clinic (437).

### Target the Genetic and Biochemical Mechanisms That Control Channel Expression

As mentioned in the introduction, peripheral nerve injury or neuropathy is associated with the generation and release of a variety of inflammatory mediators (17–20). These mediators generally increase Na^+^, Ca^2+^ and HCN channel function and attenuate K^+^ channel function (8, 24, 27, 28, 114) thereby promoting the increase in primary afferent excitability which is crucial for the onset of and persistence of neuropathic pain (11, 12, 29–35). Despite the careful documentation of changes in peripheral ion channels associated with neuropathic pain, clinical results with K^+^ channel activators and novel Na^+^ or Ca^2+^ channel blockers have met with limited success.

One possible solution is to target the processes which control the function of multiple channel types. We have already mentioned the role of the histone methyltransferase G9a in controlling the expression of K_v_7.2, K_v_1.4, and K_Ca_1.1 [(250), Table 1]. A G9a inhibitor, vorinostat is available for the management of cutaneous T-cell lymphoma. Perhaps repurposing this clinically-approved drug may lead to effective pain treatments.

The MNK-eIF4E signaling axis represents another potential drug target (110). These authors showed that a single phosphorylation site on S209 of the mRNA 5 cap-binding protein eIF4E is a critical mechanism for changes in nociceptor excitably. This is brought about by activation of mechanistic target of rapamycin (mTOR) and mitogen-activated protein kinases (MAPK) 1&2 which are downstream effectors of pro-nociceptive agents such as NGF (215) and IL-6 (476). MAPK 1 & 2 act through MAPK-interacting kinases (MNK) 1 & 2 and co-operates with mTOR to activate specific mRNA's. Nociceptor sensitization and pain behaviors are attenuated in neurons from eIF4E (S209A) mice where serine 209 is replaced by alanine, *Mnk1/2* knockout mice and by the MNK1/2 inhibitor cercosporamide. These findings underline the idea that pathways that regulate mRNA translation are key factors in changes in injury-induces nociceptor excitability and in the maintenance and/or onset of neuropathic pain These findings beg the question of whether cercosporamide, which is already used to treat and control pain in rheumatoid arthritis (477) may also be useful in other forms neuropathic pain (Table 1).

### Use the Right Combination

Combination therapy is a useful therapeutic technique that maximizes drug effects whilst limiting untoward effects. The use of low doses of two drugs that have different and possible synergistic mechanisms lessens their dose limiting side effects (478). A good example comes from the field of cardiovascular pharmacology. Both thiazide diuretics and angiotensin converting enzyme (ACE) inhibitors are useful in the management of hypertension. The combination of low doses of these drugs limits side effects. In this case, thiazides tend to lower blood K^+^ whereas ACE inhibitors tend to elevate it. In this case, the combination of drugs limits perturbation of blood K^+^ levels.

Combination therapy has been employed in pain management for many years (478), and in several cases increased therapeutic effects have been achieved using “add on” therapies which are not always based on rational application of known drug mechanisms. One logically derived combination therapy is the combination of opioids and Na_v_1.7 blockers (172) as endogenous opioids appear to be involved in their action (51, 52, 171). To best of our knowledge this type of drug combination has not yet been examined in the clinic.

### Use Human Nerves

Several recent reviews have commented on the slow translation between animal studies and the development of new therapeutic agents for use in the clinic (48, 475, 479). This reflects the self-evident differences between the human and rodent nervous systems (480). It is already known that both rodent and human and nociceptors are more heterogeneous at a molecular level than previously appreciated, and although there are broad similarities between human and rodent nociceptors there are also important differences involving ion channel function, expression, and cellular excitability (356, 479). For example, murine *SCN11A* which codes for Na_v_1.9 is only 75% identical to the human gene (114). Differences in channel structure between humans and rodents may result in differences in pharmacology. Drugs identified to work well in rodent models may be less effective in humans.

Up until recently there were few feasible methodologies available for study of human nerves. However, recent advances in technology and methodology have increased the feasibility of human studies (356, 479). For example, nociceptor morphology can be observed using biopsy samples (481) and cultured human nociceptors (482). Acutely-isolated human DRG's have been obtained from donors undergoing surgical treatment that required ligation of spinal nerve roots for spinal reconstruction or to facilitate tumor resection (12) or from organ donors (356).

Amongst other differences, this has revealed that most human DRG neurons exhibit TRPV1 receptor channels whereas in rats, it is nearly exclusively expressed in peptidergic nociceptors (483). There are also pronounced differences between HVA Ca^2+^ currents in human DRG compared to rats. Thus, in human DRG, Ca^2+^ current density is significantly smaller, kinetics of activation, inactivation, and deactivation are slower but the proportion of nifedipine-sensitive currents is far greater (356). Perhaps this relates to the report that nifedipine may be effective in management of complex regional pain syndrome (355). A further difference between human and rat DRG neurons is that a subpopulation of human neurons display relatively large constitutive Ca^2+^ current inhibition as demonstrated by paired pulse facilitation in the absence of agonist (356).

The issue of limited availability of human DRG is also being addressed using human induced pluripotent stem cells (hiPSC) and differentiating them in into nociceptive sensory neurons (54, 165, 484–486). This type of system has the advantage of scalability (generation of large numbers of cells), investigation of multiple tissue types (generation of glial and immunocompetent cells) (487) and the application of high throughput technologies such as screening of small molecule therapeutic agents and gene therapy approaches to nociceptor function (488).

Microneurography which allows i*n vivo* recording of nociceptor axonal electrical activity in humans has been available for many years (489). Technological improvements have shown that the specific C-fiber subpopulation affected (mechanoinsensitive vs. non-mechanoceptive) depends on the source of neuropathic pain and the type of neuropathy (479, 490) Modern microneurography approaches will thus play a role in the application of personalized medicine approaches to individual patients.

### Find a CRISPR Solution

There is considerable interest in the application of molecular biological approaches such as use of CRISPR (clustered regularly interspaced short palindromic repeats) technology for the management of neuropathic pain. For example, McDermott et al. (54) used CRISPR technology to edit a Na_v_1.7 mutation to restore the pain phenotype in hiPSCs from patients with congenital insensitivity to pain (CIP). As already mentioned Moreno et al. (55) recently targeted Na_v_1.7 using CRISPR-dCas9 technology by using a novel approach that prevented expression of Na_v_1.7 by editing a regulatory sequence. These authors suggested that this “LATER” (long-lasting analgesia *via* targeted *in vivo* epigenetic repression) technology might have therapeutic potential in management of persistent pain states, including primary erythromelalgia or paroxysmal extreme pain disorder. The feasibility of this type of approach has recently been reviewed (491, 492).

## Author Contributions

All authors were involved in the writing and/or review of the manuscript.

## Funding

PS was supported by Canadian Institutes of Health Grant MOP 81089 and research supplements from the Faculty of Medicine and Dentistry, University of Alberta. SA receives funding from the Research Endowment fund of the Department of Anesthesiology and Critical Care Medicine, University of New Mexico School of Medicine and a US Department of Defense Chronic Pain Management Research Program Investigator-Initiated Research Award W81XWH-20-1-0930.

## Conflict of Interest

SA is an inventor on two U.S. provisional patents (493, 494). The remaining author declares that the research was conducted in the absence of any commercial or financial relationships that could be construed as a potential conflict of interest.

## Publisher's Note

All claims expressed in this article are solely those of the authors and do not necessarily represent those of their affiliated organizations, or those of the publisher, the editors and the reviewers. Any product that may be evaluated in this article, or claim that may be made by its manufacturer, is not guaranteed or endorsed by the publisher.
